# A Meta-Analysis of Multiple Matched Copy Number and Transcriptomics Data Sets for Inferring Gene Regulatory Relationships

**DOI:** 10.1371/journal.pone.0105522

**Published:** 2014-08-22

**Authors:** Richard Newton, Lorenz Wernisch

**Affiliations:** Biostatistics Unit, Medical Research Council, Cambridge, United Kingdom; Medical University Hamburg, University Heart Center, Germany

## Abstract

Inferring gene regulatory relationships from observational data is challenging. Manipulation and intervention is often required to unravel causal relationships unambiguously. However, gene copy number changes, as they frequently occur in cancer cells, might be considered natural manipulation experiments on gene expression. An increasing number of data sets on matched array comparative genomic hybridisation and transcriptomics experiments from a variety of cancer pathologies are becoming publicly available. Here we explore the potential of a meta-analysis of thirty such data sets. The aim of our analysis was to assess the potential of *in silico* inference of *trans*-acting gene regulatory relationships from this type of data. We found sufficient correlation signal in the data to infer gene regulatory relationships, with interesting similarities between data sets. A number of genes had highly correlated copy number and expression changes in many of the data sets and we present predicted potential *trans*-acted regulatory relationships for each of these genes. The study also investigates to what extent heterogeneity between cell types and between pathologies determines the number of statistically significant predictions available from a meta-analysis of experiments.

## Introduction

The most straightforward approach to finding downstream dependent genes regulated by a candidate gene is to perform a randomisation experiment and manipulate the expression levels of that gene either by suppression or over induction. Genes which respond exclusively to the change in induced expression of the candidate gene can then be assumed to be regulated by that gene through some, possibly quite indirect, causal pathway.

However, such experiments are usually costly and time consuming to perform. In cancer cells natural randomisation might provide a substitute for explicit manipulation experiments. The variation in gene copy numbers observed in such cells provides a natural randomisation experiment. In Goh et al. [1] we experimentally validated a large proportion of regulatory pairs inferred *in silico* from matched array comparative genomic hybridisation (aCGH) and gene expression experiments, thus proving the viability and value of such an approach. The study was based on a few matched data sets only and focused on a few top ranking genes for experimental validation.

In the current study we extend the number of data sets considerably to thirty and assess whether combining data sets into a very large meta-analysis can mitigate or overcome some of the problems of inferring gene regulatory relationships from this type of data. A meta-analysis could have the capacity to increase the statistical power of predictions, but does depend on the degree of consistency that exists between data sets.

For tumor cells, aCGH microarrays compare gene copy numbers in the DNA extracted from the cells under investigation to the gene copy numbers in normal control cells, in order to detect gene deletions or gene amplifications (double or more copies of a gene compared to normal). Typically, the DNA is extracted from a tumour sample containing many cells, which may exhibit different alterations in copy number. So for each gene the measured change in copy number is an average for all the cells in the sample and will, in general, be fractional rather than integer. The gene expression experiments also utilise microarrays, but measure the abundance of mRNA.

The main purpose of this type of dual experiment is to identify potential driver genes for the cancer being studied. That is, the aCGH data is searched for genes with a known regulatory role whose copy number is altered in the samples. The matched transcriptomics data is then examined to see if a gene's altered copy number is associated with a concurrent change in the gene's expression [2]–[17], thus adding weight to the argument that the gene may be contributing to the type of cancer in question [18]. A number of algorithms and bioinformatics tools have been published to aid this type of study [17], [19]–[23]. Matched data sets have also been used for cancer subtype stratification [21], [24]–[26]. Huang et al. [18] present a useful review of past work, as do Lahti et al. [27] who compare in detail the available software packages for analysing matched data sets.

Analysis of matched data sets can however be extended to look for the potential downstream relationships of any gene in the data set which has a correlated change in aCGH and expression, not just putative oncogenic driver genes; the emphasis of the investigation going beyond cancer genetics to establishing causal gene regulatory relationships [1], [28]. By regulatory relationship we mean either a direct relationship, of a transcription factor on its target gene, or a very indirect one, through a pathway containing many intermediate regulatory steps.

Regulatory relationships can be classified as either *cis*-acting, where the regulator and target gene occur on the same chromosome and in the same region of that chromosome, and *trans*-acting where the two genes have a greater physical separation. Most studies have been concerned with *cis*-acting effects, examining how a change in copy number effects a gene's own expression and the expression of genes in the same chromosomal locus. More recently *trans*-acting effects have been investigated with the technique, with findings corroborated by gene-set enrichment and pathway analysis [29]–[31]. A very limited amount of experimental validation of predicted regulatory effects have been carried out. Akavia et al. [32] looked for driver genes and gene modules associated with these driver genes and carried out gene knockdowns followed by gene-set enrichment to validate their findings. Li et al. [28] used matched gene expression and copy number data to predict gene regulatory relationships, followed by knockdown experimental validation on a predicted regulating gene. In our recent work [1], 20 predicted regulator-target pairs, involving 5 predicted regulators, were tested experimentally by knockdown experiments. The emphasis of the study was not to identify potential oncogenes or cancer subtype stratification, rather we were using the disrupted genomes as natural knockdown, or gene copy number altering, experiments. And unlike previous studies which have analysed matched data sets in isolation, we incorporated ten matched experiments into a meta-analysis.

In this paper we perform a meta-analysis on 30 publicly available matched aCGH/expression data sets, comprising several types of cancer and a total of 2521 samples. Many genes that have altered copy number in one cancer type are found to have altered copy number in other cancer types [33], so combining data sets from multiple cancer types should help reinforce any information within the data on regulator-target relationships. In this study we concentrate on *trans*-acting relationships, since elucidating *cis*-acting relationships from matched data sets is complicated by confounding from co-amplification of regions of the genome.

The study has two aims. Firstly we document the most commonly occurring genes that have an altered copy number accompanied by a correlated change in gene expression; investigating the consistency of these correlations across cancer types and data sets. We select these genes as the most promising genes to take forward to the second part of the study where we examine the potential of using the experiments to identify *trans*-acting regulatory relationships. We chose to adopt a meta-analysis approach that highlights those gene relationships which are found in the maximum number of data sets.

## Materials and Methods

### Data

There are now a number of publicly available matched aCGH/transcriptomics experiments. Experiments were not included if they involved only a few samples, or if there was insufficient information provided to match aCGH and transcriptomics probes, or if the data covered only part of the genome. Twenty eight were selected for the meta-analysis described in this paper. The number of samples in the experiments ranged from 8 to 356. The mean number of samples was 84 and the total number of samples included in the meta-analysis was 2521. If an experiment used two different expression platforms then the samples for each expression platform were treated as a separate data set. This was done in order to avoid the possibility of spurious correlations which may be caused by systematic distortions or shifts between the two sets of expression data. This situation pertained to two of the experiments, so these two experiments contributed four data sets to the study, resulting in a total of 30 data sets. In the following we will refer to the 28 actual studies as *experiments* and the 30 sets of data derived from these experiments as *data sets*. Table 1 gives details of the 30 data sets, their size, origins and pathologies. Each of the data sets was pre-processed as follows. The aCGH data was location and scale normalized using the median and mad, as was the expression data. The aCGH and expression probes were mapped by the gene names of probes to give the maximum number of probes with corresponding aCGH and expression profiles. If necessary probe gene names were converted from synonyms to standard gene names using the database of the HUGO Gene Nomenclature Committee (HGNC) [34]. If there was more than one probe for any gene name then the median value of the probes was taken to represent that gene name. Note that the aCGH data was not thresholded so that, in general, fractional rather than integer aCGH values were used in the analysis. Fractional variations in copy number occur because of the heterogeneity of the cancer samples being studied. By using matched aCGH and expression profiles we eliminated the effects of a sample's heterogeneity considering that both sets of data were affected equally.

**Table 1:** Details of the 30 data sets used in the meta-analysis.

Code	GEO	Publication	N	P	Pathology
parr	GSE20486	Parris et al. 2010 [105]	97	18616	Breast Cancer (Diploid)
crow	GSE15134	Crowder et al. 2009 [106]	31	16153	Breast Cancer (ER+)
sirc	GSE17907	Sircoulomb et al. 2010 [107]	51	14689	Breast Cancer (ERBB2 amplified)
myll	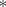	Myllykangas et al. 2008 [108]	46	17050	Gastric Cancer
junn	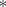	Junnila et al. 2010 [109]	10	16844	Gastric Cancer
ch.w	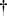	Chitale et al. 2009 [110]	91	10285	Lung adenocarcinoma
ch.s	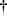	Chitale et al. 2009 [110]	94	10285	Lung adenocarcinoma
hoac	GSE20154	Goh et al. 2011 [111]	54	14388	Oesophageal adenocarcinoma
zho	GSE29023	Zhou et al. 2012 [112]	115	13697	Multiple Myeloma
shai	GSE26089	Shain et al. 2012 [7]	68	14201	Pancreatic Cancer
vain	GSE28403	Vainio et al. 2012 [16]	13	10107	Prostate Cancer
bott	GSE29211	Bott et al. 2011 [113]	53	10321	Pleural Mesothelioma
bekh	GSE23720	Bekhouche et al. 2011 [8]	173	13682	Breast Cancer (Inflammatory)
chap	GSE26863	Chapman et al. 2011 [114]	245	13667	Multiple Myeloma
ooi	GSE22785	Ooi et al. 2012 [10]	14	10091	Neuroblastoma
brag	GSE12668	Braggio et al. 2009 [115]	11	10310	Waldenströms Macroglobulinemia
jons	GSE22133	Jönsson et al. 2010 [11]	356	4183	Breast Cancer
mura	GSE24707	Muranen et al. 2011 [12]	47	4472	Breast Cancer
lin1	GSE19915	Lindgren et al. 2010 [13]	72	4965	Urothelial Carcinoma
beck	GSE17555	Beck et al. 2010 [14]	18	12174	Leiomyosarcoma
toed	GSE18166	Toedt et al. 2011 [116]	74	4289	Astrocytic Gliomas
ell	GSE35191	Ellis et al. 2012 [117]	124	13569	Breast Cancer
gra.1	GSE35988	Grasso et al. 2012 [118]	85	12849	Prostate Cancer
gra.2	GSE35988	Grasso et al. 2012 [118]	34	12813	Prostate Cancer
lenz	GSE11318	Lenz et al. 2009 [17]	203	15212	Lymphoma
lin2	GSE32549	Lindgren et al. 2012 [15]	131	8450	Urothelial Carcinoma
micc	GSE38230	Micci et al. 2013 [119]	12	16657	Vulva Squamous Cell Carcinoma
tayl	GSE21032	Taylor et al. 2010 [6]	155	14572	Prostate Cancer
coco	GSE25711 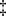	Coco et al. 2012 [120]	36	4394	Neuroblastoma
med	GSE14079	Medina et al. 2009 [121]	8	6376	Lung Cancer

GEO  =  Gene Expression Omnibus data set reference (http://www.ncbi.nlm.nih.gov/geo/), N  =  Number of samples, P  =  Number of matched probes, 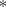 http://www.cangem.org/, 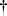 http://cbio.mskcc.org/Public/lung_array_data/, 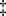 Expression data in ArrayExpress (http://www.ebi.ac.uk/arrayexpress/): E-TABM-38, E-MTAB-161.

Figure S1 in File S1 gives thirty quantile-quantile plots, one for each of the data sets, showing the Pearson correlations between a gene's aCGH profile and its expression profile for each gene in the data set. The plots demonstrate the degree to which the aCGH/expression correlations deviate from what would be expected from the correlations of two random data sets of the same size.

### Analysis

#### Overview

To perform the analysis we use the approach for analysing matched array comparative genomic hybridisation and transcriptomics experiments that we adopted in our previous study [1]. This is a relatively simple method based on correlations which provides a robust method for analysing relationships amongst large amounts of data of unknown complexities. More sophisticated network inference methods are generally much more susceptible to noise and heterogeneity between data sets. The great strength of our simple approach is that it avoids the confounding that can occur when expression data alone is used in the analysis.

We define a ‘regulating gene’ as one whose up or down expression change has a direct or indirect effect on the up or down regulation of a ‘target gene’. Primary candidates for regulating genes are genes having corresponding changes in their mRNA expression levels following copy number alterations. The regulatory relationship between regulating gene and target gene can be a direct relationship (of a transcription factor on its target gene) or a very indirect one through intermediate regulatory steps, for example the downstream transcriptional effects of genes at the top of signal transduction chains.

**Figure 1:**
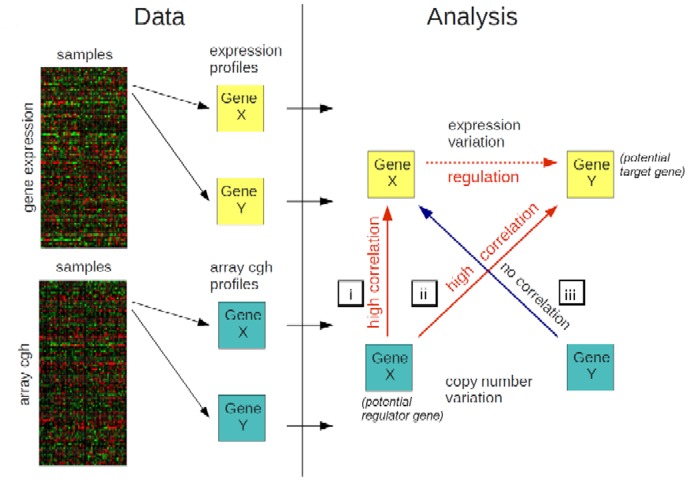
Schematic diagram illustrating the key analysis steps.

To identify potential regulator-target relationships we used three conditions: i) the correlation between the expression changes of a potential *regulating* gene with its own aCGH profile (to be worth considering as a potential regulator we are interested in those genes with a significant correlation under this condition); ii) the correlation between the expression changes of a potential *target* gene with its regulating gene's aCGH profile (here we are interested in those gene pairs with a significant correlation under this condition); iii) the correlation between a regulating gene's expression changes and its potential target gene's aCGH profile (here we require the correlations not to be significant). We used the outcome from statistical tests of these three correlations to rank the probability of a regulatory relationship for all gene pairs. Figure 1 illustrates the steps involved in the analysis. Analysis was performed using the R statistical environment [35]. The analysis code in R can be found in Goh et al. [1].

The last step, iii), is required since copy number variation may not only affect the coding sequence for one gene but possibly many genes in the neighbourhood on a genome level. In this case it would be impossible to say whether an aCGH/expression correlation between two genes is due to a regulatory affect or simply due to the two genes having similar aCGH profiles. Criterion iii excludes the possibility that the target gene is within such a neighbourhood. In this study however we were interested in *trans*-acting relationships only so this final step is of less importance. We defined *trans*-acting regulation to mean that the regulator and target are on different chromosomes. We used this definition for computational simplicity and speed, although other definitions of *trans*-acting exist, Curtis et al. [31] for example define this as a physical separation of more than 3-Megabases (in the discussion we show that using an alternative definition of *trans*-action would make only a small difference to the results).

Here we first describe the methods adopted for identifying potential regulators and assessing the consistency of these predictions. We then desecribe how we identify potential regulator-target relationships for the regulators found in the first step, and how we assess the consistency of these predictions between data sets.

#### Identifying potential regulators

In order to identify potential regulators, suitable for our three-step approach to identifying regulatory pairs, we focus on genes with a high correlation between their copy number and their gene expression. Various correlation measures could be applied. Partial correlation might be suggested in order to mitigate confounding effects from genes with similar copy number changes to the candidate gene through, for example, vicinity in the genome. Nonparametric measures of correlation, such as Spearman rank correlation, might be more robust than Pearson correlation for highly nonlinear, non Gaussian data. We performed a comparison of various correlation measures based on cross-validation (see File S1) and found Spearman correlation to be the most consistent, we therefore use it throughout the rest of this paper.

In the first instance, 30 Spearman rank correlations (from the 30 data sets), and their *p*-values for being greater than zero, were calculated for each gene (R function cor.test). These 30 *p*-values were combined for each gene into a single *p*-value statistic using Fisher's method (R function survcomp::combine.test). In order not to rely on any statistical assumptions we obtained a null distribution of combined *p*-value statistics through permutation of gene identifiers (see below). The resulting *p*-values for each gene were finally corrected for multiple testing by the Benjamini-Hochberg (B-H) method, to give a false discovery rate (fdr) for each gene based on its aCGH/expression correlations in the 30 data sets. In the following the Benjamini-Hochberg adjusted *p*-values are referred to as B-H adjusted *p*-values and are now fdr values rather than *p*-values in the sense of a type I error.

We were also interested in how many, and which, of the 30 data sets indicated an aCGH/expression correlation. This was assessed for each of the genes using an arbitrary threshold of 0.05 on a gene's 30 correlation *p*-values after adjustment for multiple testing.

To generate the null distribution, 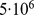 permutations of gene identifiers were generated for each data set and the above procedure, using Fisher's method, for obtaining combined *p*-value statistics repeated. In practice only a minority of genes are present in all 30 data sets. In general a gene will be present in less than 30 data sets, hence we generated 30 null distributions for *n* combined *p*-values, *n* from 1 to 30.

The consistency of potential regulator predictions were tested both *within* each data set and *between* data sets. For *between* data set consistency, for each data set we obtained a list of genes ordered by their fdr for significant correlation. We also obtained a subset of top-ranking genes with an fdr of less than 0.05. The Kolmogorov-Smirnov test was used to test the top-ranking genes derived from one data set for enrichment in the ordered gene list derived from a second data set. *p*-values for enrichment were calculated by permutations of gene identifiers.

For *within* data set consistency each dataset was studied independently. A data set was randomly divided into two equal sized data sets, and two lists of correlation *p*-values were calculated from each of these, ordered by increasing *p*-value. The correlation being between each gene's aCGH profile and its expression profile. In order to compare the two lists, one approach would be a rank correlation method such as Kendall's 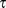. The lists to be compared are however very long and in practice we are interested in only the top most significant genes, but Kendall's 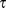 places equal weight on the rankings of genes anywhere in the list. We therefore adopted a method which takes the top genes in one list (a gene-set) and looks at their ranks in the second list, and vice-versa. In order to treat all datasets equally in this comparison analysis we took the top genes to be the top *s* genes in a list in all cases. The size of the gene-set *s* was arbitrarily chosen to be 10. The Kolmogorov-Smirnov test (R function ks.test) was used to test whether the gene-set derived from the first half of the dataset was enriched in the ordered list from the second half of the dataset. For each dataset this procedure was repeated ten times, that is, on ten random divisions of the dataset. The result was a mean and range of cross-validation enrichment scores for each dataset. *p*-values for enrichment were calculated by permutations of gene identifiers.

#### Regulator-target relationships

After we found potential regulators fulfilling condition i) of our three criteria we looked for potential target genes of these regulators applying criterion ii) expression changes of a potential target gene must correlate highly with its regulating gene's aCGH profile and criterion iii) the correlation between a regulating gene's expression changes and its potential target gene's aCGH profile must be low.

The correlation tests were similar to those in the previous section to find potential regulators but with three additions. Firstly, we tested separately the two alternative hypotheses: that the correlation of a regulator-target pair is greater than zero and that the correlation is less than zero, and we generated separate null distributions for the two conditions. Secondly, for each potential regulator only those data sets were included in the analysis for which that regulator had a significant self aCGH/expression correlation. Thirdly, since we were only interested in *trans*-acting relationships the null distributions were derived using potentially *trans*-acting gene pairs. A null distribution based on *trans*-acting pairs is required since the frequency of significant correlations is lower than for *cis*-acting pairs.

As for potential regulators the consistency of the predictions between data sets was assessed using gene-set enrichment analysis. For a given potential regulator, for each of the 30 data sets a list of potential *trans*-acted targets was generated ordered by significance of correlation with the regulator. For each data set we also obtained a subset of top-ranking genes with an fdr of less than 0.05. To compare any two data sets for consistency the set of top-ranking genes from one data set was tested for enrichment in the complete ordered gene list of the second data set, and vice-versa, and the two *p*-values averaged.

Just because a gene appears in a regulator's list of predicted targets, does not mean that regulator is the most probable regulator for that target. Therefore, for each of the top potential regulators, all predicted *trans*-acted targets were removed if the data indicated an alternative, more probable, regulator. This procedure was found to be important, reducing the number of predicted targets in most cases.

## Results

### Potential regulators

Table 2 lists the top 30 potential regulators excluding known transcription factors, while Table 3 lists the top 30 potential regulators known to be transcription factors (according to the list of human transcription factors from the Transfac database [36], [37]). The genes in the table are ordered by the number of data sets which indicate a significant correlation (B-H adjusted *p*-value <0.05), so as to highlight the potential regulators which are significant in the largest number of different pathologies. Sheet S1 in File S2 gives the full list of potential regulators. The list includes only those genes which have significant aCGH/expression correlation in at least one of the data sets.

**Table 2:** Top 30 potential regulators - not transcription factors, based on the Spearman correlation of a gene's aCGH with its expression, from a meta-analysis of the 30 data sets.

Gene	Chr	Locus	*p*-value	N	Annotation
PCM1	8	22-p	5.9e-05	17	Pericentriolar Material 1
ELP3	8	21.1p	5.9e-05	17	Elongator Acetyltransferase Complex Subunit 3
MED4	13	14.12q	5.9e-05	17	Mediator complex subunit 4
MCPH1	8	23.1p	5.9e-05	16	Microcephalin 1
COPS3	17	11.2p	0.0087	16	COP9 constitutive photomorphogenic homolog subunit 3
PREP	6	22q	5.9e-05	15	Prolyl endopeptidase
DDX10	11	22-q	5.9e-05	15	DEAD (Asp-Glu-Ala-Asp) box polypeptide 10
BCL9	1	21q	5.9e-05	15	B-cell CLL/lymphoma 9
CDC16	13	34q	5.9e-05	15	Cell division cycle 16
HDAC2	6	21q	5.9e-05	15	Histone deacetylase 2
AZIN1	8	21.3q	5.9e-05	15	Antizyme inhibitor 1
SS18L1	20	13.3q	5.9e-05	14	Synovial sarcoma translocation gene on chromosome 18-like 1
TGDS	13	32.1q	5.9e-05	14	TDP-glucose 4,6-dehydratase
YTHDF1	20	13.33q	5.9e-05	14	YTH domain family, member 1
COG2	1	42.2q	5.9e-05	14	Component of oligomeric golgi complex 2
PPP2R2A	8	21.2p	5.9e-05	14	Protein phosphatase 2, regulatory subunit B, alpha
PTDSS1	8	22q	5.9e-05	14	Phosphatidylserine synthase 1
AKAP11	13	14.11q	5.9e-05	14	A kinase (PRKA) anchor protein 11
IKBKB	8	11.2p	5.9e-05	14	Inhib. of kappa light polyp. gene enhancer in B-cells, kinase beta
MBTPS1	16	24q	5.9e-05	14	Membrane-bound transcription factor peptidase, site 1
UCHL3	13	21.33q	5.9e-05	14	Ubiquitin carboxyl-terminal esterase L3 (ubiquitin thiolesterase)
AARS	16	22q	5.9e-05	14	Alanyl-tRNA synthetase
ATXN10	22	13q	5.9e-05	14	Ataxin 10
RAF1	3	25p	5.9e-05	14	V-Raf-1 murine leukemia viral oncogene homolog 1
PPP3CC	8	21.3p	5.9e-05	14	Protein phosphatase 3, catalytic subunit, gamma isozyme
TBCE	1	42.3q	5.9e-05	14	Tubulin folding cofactor E
RIPK2	8	21q	0.0087	14	Receptor-interacting serine-threonine kinase 2
INTS6	13	14.3q	0.0087	14	Integrator complex subunit 6
UBAP2	9	11.2p	0.0087	14	Ubiquitin associated protein 2
GNA12	7	22.3p	0.0087	14	Guanine nucleotide binding protein (G protein) alpha 12

Chr  =  Chromosome, Locus  =  Gene locus, *p*-value  =  B-H adjusted *p*-value, N  =  number of data sets with significant correlation (B-H adjusted *p*-value <0.05).

**Table 3:** Top 30 potential regulators - transcription factors, based on the Spearman correlation of a gene's aCGH with its expression, from a meta-analysis of the 30 data sets.

Gene	Chr	Locus	*p*-value	N	Annotation
GTF2F2	13	14q	5.9e-05	16	General transcription factor IIF, polypeptide 2
TAF2	8	24q	5.9e-05	14	TATA box binding protein (TBP)-associated factor
SETDB1	1	21q	5.9e-05	14	SET domain, bifurcated 1
ELF1	13	13q	0.0087	14	E74-like factor 1 (ets domain transcription factor)
YWHAZ	8	22.3q	5.7e-05	13	Tyrosine/tryptophan activation protein, zeta polypeptide
PARP1	1	41-q	0.0087	13	Poly (ADP-ribose) polymerase 1
ACTL6A	3	26.33q	0.0087	13	Actin-like 6A
PSMB1	6	27q	0.0087	13	Proteasome subunit, beta type, 1
SMARCA2	9	24.3p	0.0087	13	SWI/SNF related, matrix associated, actin dependent regulator of chromatin, subfamily a, member 2
NCOR1	17	11.2p	0.0087	13	Nuclear receptor corepressor 1
MAP3K7	6	15q	0.0087	13	Mitogen-activated protein kinase kinase kinase 7
HSBP1	16	23.3q	5.7e-05	12	Heat shock factor binding protein 1
SMARCE1	17	21.2q	5.9e-05	12	SWI/SNF related, matrix associated, actin dependent regulator of chromatin, subfamily e, member 1
POGZ	1	21.1q	5.9e-05	12	Pogo transposable element with ZNF domain
RCOR3	1	32.3q	5.9e-05	12	REST corepressor 3
TRIM33	1	13.1p	5.9e-05	12	Tripartite motif containing 33
ARID4B	1	42.1-q	5.9e-05	12	AT rich interactive domain 4B (RBP1-like)
MNAT1	14	23q	5.9e-05	12	Menage a trois homolog 1, cyclin H assembly factor (X. laevis)
NFATC3	16	22q	5.9e-05	12	Nucl. factor of activated T-cells, cytoplasmic, calcineurin-dep. 3
TBP	6	27q	5.9e-05	12	TATA box binding protein
AATF	17	12q	5.9e-05	12	Apoptosis antagonizing transcription factor
SMAD2	18	21q	5.9e-05	12	SMAD family member 2
AP2B1	17	11.2-q	0.0087	12	Adaptor-related protein complex 2, beta 1 subunit
SNAPC3	9	22.3p	0.0087	12	Small nuclear RNA activating complex, polypeptide 3
SNW1	14	22.1-q	0.0087	12	SNW domain containing 1
SMARCC1	3	21.31p	0.0087	12	SWI/SNF related, matrix associated, actin dependent regulator of chromatin, subfamily c, member 1
HSF2	6	22q	0.0087	12	Heat shock transcription factor 2
PSIP1	9	22.2p	0.0087	12	PC4 and SFRS1 interacting protein 1
RB1	13	14.2q	0.0087	12	Retinoblastoma 1
CREBBP	16	13.3p	0.0087	12	CREB binding protein

Chr  =  Chromosome, Locus  =  Gene locus, *p*-value  =  B-H adjusted *p*-value, N  =  number of data sets with significant correlation (B-H adjusted *p*-value<0.05).

**Figure 2:**
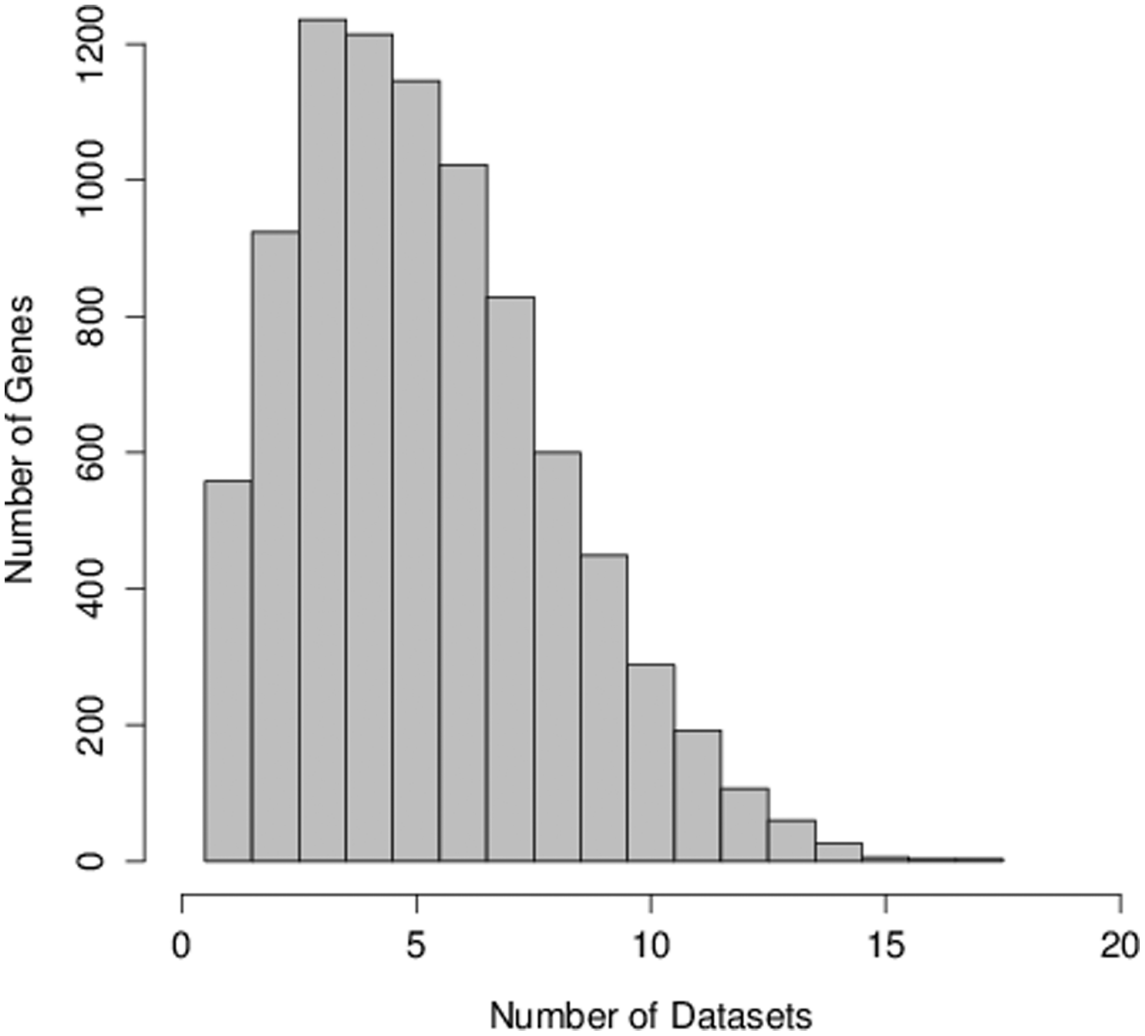
Histogram showing the number of genes which are potential regulators in different numbers of data sets. For each gene the number of individual data sets in which the Spearman correlation between a gene's aCGH and expression has an B-H adjusted *p*-value <0.05 is counted. The graph shows a histogram of these counts. Only those genes which have a *combined* B-H adjusted *p*-value <0.05 are included in the histogram.

Figure 2 shows a histogram of the number of potential regulators detected in different numbers of data sets. For all potential regulators, that is those genes which have a *combined* B-H adjusted *p*-value <0.05, the number of individual data sets in which the gene's aCGH/expression correlation has a B-H adjusted *p*-value <0.05 is counted. The graph shows a histogram of these counts. The maximum number of data sets in which genes show significant aCGH/expression correlation is 17, and rather few genes are found with this maximum count. The majority of potential regulators have significant correlation in a relatively small subset of the 30 data sets.

Examining the results for PCM1, the gene at the top of Table 2. PCM1 has significant aCGH/expression correlation in 17 of the 30 data sets. Of the 13 data sets in which it did not show significant aCGH/expression correlation at a B-H adjusted *p*-value threshold of 0.05, the gene was not annotated in 2 data sets, it was close to significant in one data set (B-H adjusted *p*-value  = 0.051) and had a B-H adjusted *p*-value <0.15 in 3 data sets. We examined the remaining 7 data sets to see whether the lack of significant aCGH/expression correlation was because PCM1 did not show copy number variation in these data sets, or because it did show copy number variation but this was not correlated with its expression. To assess copy number variation in a data set we measured the variance of all the genes in the data set and took the mode of the distribution of the variance as an arbitrary threshold for copy number variation. Using this criterion, for PCM1, 4 of the 7 data sets which had no significant aCGH/expression correlation did show copy number variation and 3 showed no copy number variation.

**Figure 3:**
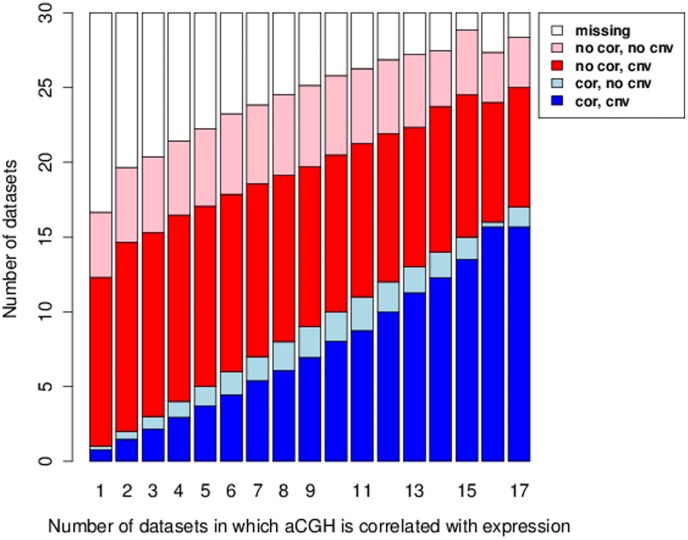
Breakdown of potential regulators in terms of number of data sets with & without aCGH/expression correlation and with & without copy number variation. Genes have been grouped according to the number of data sets in which they displayed significant aCGH/expression correlation (so from 1 data set to the maximum of 17 data sets). These groups are displayed along the horizontal axis. For each group the following five averages were calculated and displayed in the graph: 1. The average number of data sets where genes are not annotated (white bars). 2. The average number of data sets where genes do not have significant aCGH/expression correlation and do not show copy number variation (pink bars). 3. The average number of data sets where genes do not have significant aCGH/expression correlation but do show copy number variation (red bars). 4. The average number of data sets where genes have significant aCGH/expression correlation and no copy number variation (light blue bars). 5. The average number of data sets where genes have significant aCGH/expression correlation and copy number variation (dark blue bars). Were presence of copy number variation defined by the arbitrary threshold discussed in the text.

We repeated this analysis for all the genes in the study, first grouping the genes by the number of data sets in which they displayed significant aCGH/expression correlation (so from 1 data set to the maximum of 17 data sets), and then calculating five different averages for each of these 17 groups. Figure 3 shows the averages for the groups. The five average values displayed by the graph are:

- The average number of data sets where genes are not annotated.
- The average number of data sets where genes do not have significant aCGH/expression correlation and do not show copy number variation (with copy number variation defined by the arbitrary threshold discussed above).
- The average number of data sets where genes do not have significant aCGH/expression correlation but do show copy number variation.
- The average number of data sets where genes have significant aCGH/expression correlation and no copy number variation.
- The average number of data sets where genes have significant aCGH/expression correlation and copy number variation.

The graph shows that on average genes have no significant aCGH/expression correlation in around a third of the data sets despite having copy number variation in those data sets (red bars in Figure 3). The number of data sets where a gene shows no significant aCGH/expression correlation and no copy number variation is much less and fairly constant at around 4 or 5 data sets (pink bars in Figure 3). A lack of copy number variation in a data set can occur for two reasons. Firstly the gene could have no amplifications or deletions in any of the samples in the data set. Secondly it could be consistently amplified, or deleted, in all the samples in the data set.

For each data set we calculated the percentage of genes that have significant aCGH/expression correlation (B-H adjusted *p*-value <0.05) and also have copy number variation. The median value for the 30 data sets is 13% with a maximum of 63%. The values calculated in this manner are in line with those reported in the literature, namely transcriptional changes for 10–63% of genes in amplified regions and 14–62% in regions of loss, across multiple cancer types [18].

We examined the pathologies in which potential regulators show significant aCGH/expression correlation. Table S1 in File S1 lists the top 30 potential regulators (not transcription factors) and summarises in which pathologies the genes have significant aCGH/expression correlation. For Breast cancer 6 genes had significant aCGH/expression correlation at a level of 0.05 in all 7 breast cancer data sets (BCL9, AZIN1, TAF2, YTHDF1, TTC13, FBXL20). At a significance level of 0.2 this rises to 103 genes. Table S2 in File S1 is a similar table, but for the top 30 genes which are transcription factors. Sheet S2 in File S2 gives the complete list. The list includes only those genes which have significant aCGH/expression correlation in at least one of the data sets. Of the top 30 potential regulators in Table 2, nine occur on chromosome 8 and six on chromosome 13.

#### Consistency of predictions

**Figure 4:**
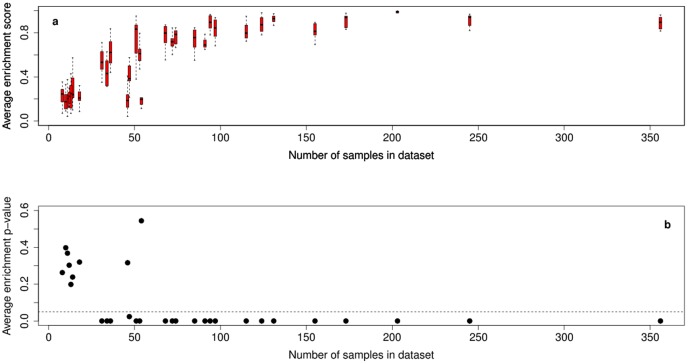
Boxplot showing the within data set cross-validation consistency. For the 30 data sets (a) enrichment scores and (b) average B-H adjusted *p*-values of enrichment scores. Each data set was randomly halved. Spearman correlation of genes' aCGH and expression values was used to rank genes in each half data set. The top 10 from the first half was used as a gene-set and scored for enrichment in the second half. This was repeated for 10 random divisions of each data set.

Figure 4 shows a boxplot of the *within* data set cross-validation enrichment scores for each of the 30 data sets. Those data sets containing large numbers of samples have high enrichment scores. In total 21 of the 30 data sets show significant within data set consistency. The data sets with few samples have rather low enrichment scores. These low values suggest that the smaller data sets may have limited predictive value. The consistency of predictions *between* data sets was tested using only the 21 data sets which showed significant within data set consistency. Of the resulting 210 B-H adjusted *p*-values, 189 were less than 0.05.

**Figure 5:**
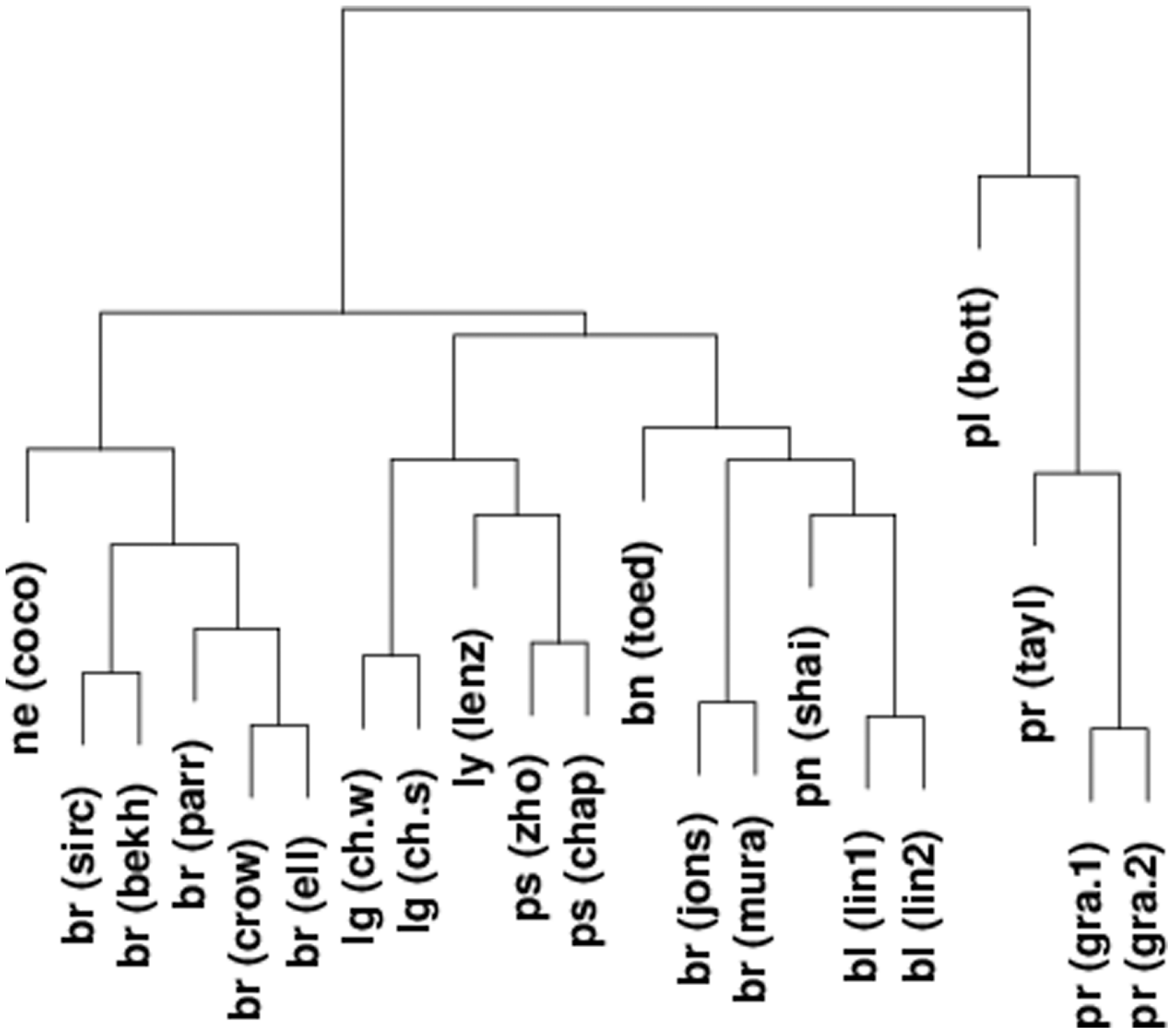
Clustering data sets according to enrichment scores. Spearman correlation of genes' aCGH and expression values was used to rank genes in each data set. The significant genes from one data set was used as a gene-set and scored for enrichment in the second half, and vice-versa. The two enrichment scores were averaged and this value minus one used as a distance measure for clustering, using Ward's method. The nine data sets with low within data set consistency were excluded from the clustering (pr = prostate, lg = lung, oa = oesophageal, ly = lymphoma, bl = bladder, br = breast, ne = neuroblastoma, pl = pleural, ps = myeloma, pn = pancreas, ga = gastric, bn = glioma).

Figure 5 shows how the 21 data sets cluster using one minus the enrichment scores *between* data sets as a distance measure and using Ward's clustering method. The different breast cancer data sets cluster together (apart from two of the breast cancer data sets), as do the two different myeloma data sets and the prostate data sets. Figure S4 in File S1 shows a similar plot but instead of ranking genes by their aCGH/expression correlation the genes were ranked by their aCGH variance. Comparing Figure 5 with Figure S4 shows that aCGH/expression correlation clusters the various pathologies better than just aCGH variance.

#### Biological context

On chromosome 8, PCM1 Pericentriolar Material 1 encodes a protein which is a component of centriolar satellites, electron dense granules found around centrosomes. The protein is essential for the correct localization of several centrosomal proteins, and for anchoring microtubules to the centrosome. Aberrations involving this gene have been found in papillary thyroid carcinomas, atypical chronic myeloid leukemia and T-cell lymphoma [38]. A fusion of PCM1 and JAK2 is a recurrent abnormality in chronic and acute leukemia [39]. PCM1 has significant aCGH/expression correlation in the breast, myeloma, lymphoma, prostate, urothelial, lung, pancreatic and neuroblastoma data sets but not in oesophageal, mesothelioma or gastric (and not annotated in glioma). ELP3, also on chromosome 8 and at close locus to PCM1, is the catalytic subunit of the histone acetyltransferase elongator complex, which contributes to transcript elongation and also regulates the maturation of projection neurons [38]. ELP3 has been identified as a signature for hepatocellular carcinoma progression [40] and has been linked to poor prognosis in endometrioid adenocarcinoma [41]. MCPH1, Microcephalin I, encodes a DNA damage response protein and is a potential tumour suppressor [42], [43]. Low levels of MCPH1 were found in chronic myeloid leukemia cells [44], correlates with survival in ovarian cancer [45] and is a prognostic indicator in breast cancer [46]–[48]. AZIN1, anitzyme inhibitor 1, regulates cellular polyamine homeostasis. Increased expression was found in prostate cancer cells [49] and RNA editing predisposes to hepatocellular carcinoma [50].

MED4 Mediator Of RNA Polymerase II Transcription, Subunit 4 Homolog (S. Cerevisiae) encodes a component of the Mediator complex, which interacts with DNA-binding gene-specific transcription factors to modulate transcription by RNA polymerase II [38]. MED4 has been associated with carcinogenesis and chemoradioresistance in cervical cancer [51]. Close to MED4 on chromosome 13, GTF2F2 is a general transcription initiation factor that binds to RNA polymerase II and helps to recruit it to the initiation complex. CDC16 encodes a component of the APC complex, which is a cyclin degradation system that governs exit from mitosis [38] and has been with an altered risk of breast cancer [52].

COPS3 encodes a protein with kinase activity that phosphorylates regulators involved in signal transduction and has found to be a potential oncogene in osteosarcoma [53], multiple myeloma [54] and lung cancer [55]. PREP, encodes a cytosolic prolyl endopeptidase and has been associated with neoplasms in an number of tissues [56]–[58]. HDAC2 encodes a protein that forms transcriptional repressor complexes playing an important role in transcriptional regulation [38], and in cancer [59]. DDX10 is a putative RNA helicases that may be involved with ribosome assembly. It has been suggested as an oncogene in breast cancer [60] and plays a role in acute myeloid leukemia as a fusion gene with NUP98 [61]. BCL9 is involved in signal transduction through the Wnt pathway and is known to promote tumour progression [62].

Looking at the top transcription factors, TAF2 RNA Polymerase II, TATA Box Binding Protein (TBP)-Associated Factor has significant aCGH/expression correlation in 14 of the data sets. YWHAZ Tyrosine 3-Monooxygenase/Tryptophan 5-Monooxygenase Activation Protein, Zeta Polypeptide belongs to the 14-3-3 family of proteins which mediate signal transduction [38] and has been suggested as having pivotal role in tumour cell proliferation [63], [64].

ELF1, E74-Like Factor 1 encodes an E26 transformation-specific related transcription factor, and has been shown to help predict the malignant behaviour of non-small cell lung cancer [65], has been associated with gastric cancer [66], [67] and with endometrial cancer [68] and may modulate breast cancer progression [69]. NCOR1 Nuclear Receptor Corepressor 1 mediates transcriptional repression by certain nuclear receptors, and has a known role in cancer [70], being associated with breast cancer [71], esophageal cancer [72] and prostate cancer [73]. PSMB1, Proteasome Subunit Beta and MAP3K7, Mitogen-Activated Protein Kinase Kinase Kinase 7, both have significant aCGH/expression correlation in 13 data sets. SETDB1, SET Domain, Bifurcated 1 regulates histone methylation, gene silencing, and transcriptional repression. It contributes to human lung tumorigenesis [74] and is recurrently amplified in melanoma [75]. PARP1, Poly (ADP-Ribose) Polymerase 1 modifies nuclear proteins by poly(ADP-ribosyl)ation. It is overexpressed in a number of cancers, and has been associated with overall prognosis in cancer [76]. ACTL6A Actin-Like 6A is significantly correlated in 13 data sets. On chromosome 9 SMARCA2, SWI/SNF Related, Matrix Associated, Actin Dependent Regulator Of Chromatin, Subfamily A, Member 2 is part of the complex that is critical for differentiation and proliferation so has been associated with malignant transformation [77].

### Inferring *trans*-acting gene regulatory relationships

Lists of potential *trans*-acted targets were generated for all the potential regulators presented in Table 2 (that is, the top 30 potential regulators which are not known to be transcription factors), as described in the ‘Methods’ section. Lists were also generated for the top 72 potential regulators which are known to be transcription factors (the top 30 of the 72 features in Table 3). For each potential regulator two lists were generated, one for positive regulatory relationships and one for negative regulatory relationships. The gene lists can be found in File S3 (positive, for top 30 that are not transcription factors), File S4 (negative, for top 30 that are not transcription factors), File S5 (positive, for top 72 which are known transcription factors) and File S6 (negative, for top 72 which are known transcription factors). Potential regulators are only included in the files if they have at least one significant predicted target (B-H adjusted *p*-value <0.1).

**Figure 6:**
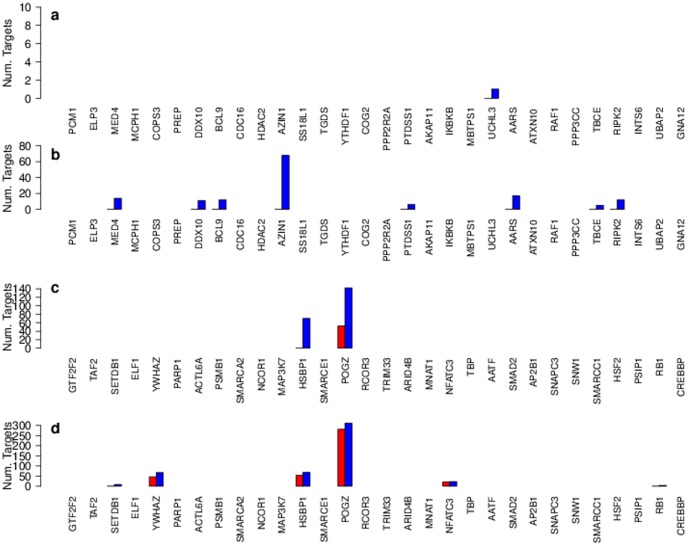
Bar charts showing the number of predicted targets for each potential regulator. At a significance level of 0.05 (red) and 0.1 (blue) a. positive regulation, top 30 potential regulators which are not transcription factors (TF) b. negative regulation, top 30 potential regulators which are not TF c. positive regulation, top 30 potential regulators which are TF d. negative regulation, top 30 potential regulators which are TF.

Figure 6 summarises the results, showing the number of significant positive and negative *trans*-acting relationships for each of the potential regulators (the figure only includes the top 30 potential regulators which are known to be transcription factors, for the remaining 42 see Figure S5 parts a & b in File S1). Many of the potential regulators have no significant predicted *trans*-acted targets. It can be seen from the graphs that the potential regulators which are transcription factors have in general more predicted relationships than the potential regulators which are not known to be transcription factors. In addition negative regulation shows more predicted targets than positive regulation.

**Figure 7:**
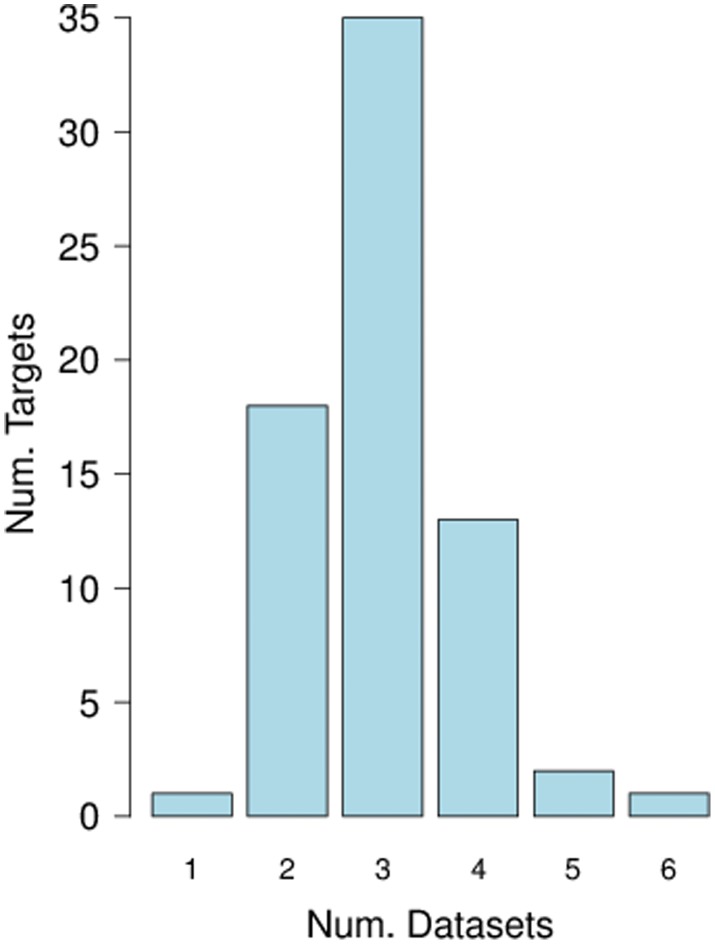
Histogram plotting the number of predicted targets which are significant. (B-H adjusted *p*-value <0.1) in different numbers of data sets for HSPB1.

Figure 7 shows for one regulator (HSBP1) how many data sets are contributing to its predicted targets. The histogram plots the number of predicted targets (B-H adjusted *p*-value <0.1) for the regulator which are significant in different numbers of data sets. In general a regulator-target pair demonstrates a significant regulator-target aCGH/expression correlation in rather few data sets.

We investigated whether the type of meta-analysis we have adopted, that is using significance level thresholds, was over-emphasising the heterogeneity of the data. To do this we examined, for each regulator, how a gene-set comprising the significant targets predicted by the *meta-analysis*, was enriched in the ranked lists of genes obtained when the 30 data sets were analysed *individually*. Table 4, displays the results. The table contains data for all the potential regulators shown in Figure 6 and Figure S5 in File S1 which have at least one predicted target from the meta-analysis at a significance level of 0.1 (marked by the blue bars in the figures).

**Table 4:** For each regulator, comparing percentage of data sets which, when analysed *individually*, predict at least one of the targets that are predicted by the *meta-analysis*, with percentage of data sets in which the gene-set of targets that are predicted by the *meta-analysis* has significant enrichment in the individual data sets' ranked lists of genes.

Gene	data sets	% Containing	% Enriched
Positive (not TF)			
UCHL3	14	21	14
Negative (not TF)			
MED4	17	47	53
DDX10	15	47	33
BCL9	15	40	33
**AZIN1**	**15**	**47**	**93**
PTDSS1	14	29	50
AARS	14	43	57
TBCE	14	29	14
RIPK2	14	29	64
Positive (TF)			
HSBP1	12	58	58
POGZ	12	58	100
SMAD5	10	70	70
Negative (TF)			
SETDB1	14	36	36
YWHAZ	13	46	69
HSBP1	12	75	75
POGZ	12	67	91
NFATC3	12	50	50
RB1	12	33	58
E2F5	11	36	55
ADAR	11	18	18
SMAD5	10	60	70
NCOA6	10	20	20
ARNT	10	50	80

data sets  =  number of data sets in which the regulator shows significant correlation between its own aCGH and expression, % Containing  =  percentage of data sets which, when analysed *individually* predict at least one of the targets that are predicted by the *meta-analysis*, % Enriched  =  percentage of data sets in which the gene-set of targets that are predicted by the *meta-analysis* has significant enrichment in the individual data sets' ranked lists of genes, TF  =  Transcription Factor.

The first column in the table gives the number of data sets in which the regulator shows significant self aCGH/expression correlation. The values in the second and third columns are expressed as percentages of this number of data sets. The second column shows the percentage of these data sets which, when analysed *individually*, predict at least one of the targets that are predicted by the *meta-analysis*. The percentages range between 18% and 75%, with a mean of 43%, so for most regulators, more than half the data sets which show significant self aCGH/expression correlation predict none of the targets predicted by the meta-analysis. The third column shows the percentage of data sets in which the *meta-analysis* gene-set of predicted targets has significant enrichment (B-H adjusted *p*-value <0.05) in the *individual* data sets' ranked lists of genes.

Comparing columns 2 and 3 of Table 4, for some regulators, only a minority of the data sets call any of the meta-analysis predicted targets as significant (column 2), but as a gene-set the meta-analysis predicted targets are significantly enriched in a far higher proportion of the data sets (column 3). For example for AZIN1 (negative regulation), Table 4 column 2 shows that 47% of the data sets, for which AZIN1 shows significant self aCGH/expression, predict none of the targets predicted by the meta-analysis, but Table 4 column 3 shows that almost all these data sets (93%) have significant enrichment of the meta-analysis list of predicted targets.

#### GO, Pathway and Citation Corroboration

We investigated to what degree publicly available data on gene regulatory relationships substantiated the predicted regulator-target pairs. The results are summarised in Table 5.

**Table 5:** Supporting evidence for regulator-target predictions.

Regulator	N. of Tg.	Co-Cites	Tg. Co-Cites	Enriched GO annotations	GO *q*-value	Enriched Pathways	Path. *q*-value
*Positive not TF*							
UCHL3	1		n/a	-			
*Negative not TF*							
MED4	14	[84]	1 (2)	GO:0065004 protein-DNA complex assembly	0.153 (2/97)	Resolution of Sister Chromatid Cohesion (R)	0.03 (2/77)
DDX10	11	[85]	4 (2)	GO:0048858 cell projection morphogenesis	0.001 (5/350)	EGFR downregulation (R)	0.005 (2/13)
BCL9	12		1 (2)	GO:0002683 negative regulation of immune system process	0.01 (3/91)	-	-
AZIN1	68		68 (5)	GO:0005515 protein binding	0.004 (44/3651)	ALK1 signaling events (P)	0.002 (4/20)
PTDSS1	6		3 (2)	GO:0033627 cell adhesion mediated by integrin	0.006 (2/28)	-	-
AARS	17		3 (2)	GO:0033059 cellular pigmentation	0.03 (2/14)	NGF signalling via TRKA from the plasma membrane (R)	0.04 (2/95)
TBCE	5		0	GO:0000226 microtubule cytoskeleton organization	0.04 (2/116)	-	-
RIPK2	12	[86]	1 (2)	GO:0030097 hemopoiesis	0.03 (4/302)	Class B/2 (Secretin family receptors) (W)	0.03 (2/43)
*Positive TF*							
HSBP1	70		216 (4)	GO:0002697 regulation of immune effector process	0.008 (8/154)	Primary immunodeficiency - H. sapiens (K)	0.007 (4/24)
POGZ	142	[87]-[91]	339 (6)	GO:0019222 regulation of metabolic process	1.25e-06 (77/2287)	Mismatch repair - H. sapiens (K)	0.02 (4/15)
SMAD5	25		2 (2)	-	-	Host Interactions of HIV factors (R)	0.04 (2/26)
*Negative TF*							
SETDB1	8		0	GO:0048589 developmental growth	0.14 (2/140)	-	-
YWHAZ	67	[92]-[94]	19 (3)	GO:0005085 guanyl-nucleotide exchange factor activity	0.03 (5/103)	Alpha4 beta1 integrin signaling events (P)	0.06 (3/23)
HSBP1	68		51 (4)	GO:0019058 viral infectious cycle	0.03 (7/143)	Apoptotic execution phase (R)	0.06 (3/26)
POGZ	311	[87], [88], [91]	650 (9)	GO:0044419 interspecies interaction between organisms	0.0002 (28/256)	Phagosome - Homo sapiens (K)	0.003 (15/84)
NFATC3	23	[95]	4 (2)	GO:0022604 regulation of cell morphogenesis	0.006 (5/143)	Fc-epsilon receptor I signaling in mast cells (P)	0.001 (3/24)
RB1	4		0	GO:0036211 protein modification process	0.002 (5/1278)	miR-targeted genes in epithelium - TarBase (W)	0.005 (2/131)
E2F5	15	[96]	38 (3)	GO:0034329 cell junction assembly	0.06 (3/123)	Integrin cell surface interactions (P)	0.002 (3/45)
ADAR	1	[97]-[100]	n/a	GO:0034097 response to cytokine stimulus	0.02 (2/276)	-	-
SMAD5	24	[101]	3 (3)	GO:0065008 regulation of biological quality	0.03 (11/1159)	Cytosolic sensors of pathogen-associated DNA (R)	0.01 (2/16)
NCOA6	1		n/a	-	-	-	-
ARNT	22		6 (3)	GO:0009057 macromolecule catabolic process	0.002 (8/450)	HIF-2-alpha transcription factor network (P)	0.04 (2/17)

TF  =  Transcription Factor; N of Tg.  =  Number of Predicted Targets at a fdr significance level of 0.05; CoCites  =  Papers which co-cite both Regulator and a predicted target, from PubMed (http://www.ncbi.nlm.nih.gov/pubmed/) using Bioconductor package org.Hs.eg.db version 2.9.0 [80] restricted to papers with less than 150 gene links, and also from manual search of PMC (http://www.ncbi.nlm.nih.gov/pmc/); Tg. Co-Cites  =  Number of papers that cite at least two of the predicted targets, with (in brackets) the maximum number of targets in any one paper, from PubMed using Bioconductor package org.Hs.eg.db version 2.9.0 [80] restricted to papers with less than 150 gene links; Enriched GO annotations and Pathways using ConsensusPathDB [81]–[83] (R  =  Reactome, W  =  WikiPathways, P  =  Pathway Interactions Database, K  =  Kegg), with *q*-values and (in brackets) the number of genes from list (composed of a regulator and its predicted targets) in the GO annotation or pathway and the total number of genes in the GO annotation or pathway.

Firstly, for each potential regulator studied that has significant predicted targets (22 in total), we looked for publications which co-cited both the regulator and any of its predicted targets. For this we used the PubMed [78] information contained in Bioconductor [79] package org.Hs.eg.db [80] (version 2.9.0). We found 9 of the regulators had such co-citations. We also looked for any publications that co-cited any two or more of a regulator's predicted targets. Most of the regulators did have co-cited predicted targets, although in most cases only two or three of the predicted targets were co-cited in any one paper. We then looked at enriched Gene Ontology (GO) annotations in the lists of predicted targets (plus their proposed regulator) using ConsensusPathDB [81]–[83]. Most of the lists had significantly enriched Biological Process GO annotations, and most at level 3 or 4. The number of genes in a list that were included together in a GO annotation ranged from 7% to 100%, with the mean being 35%. Many of the lists were also associated with significantly enriched pathways. In general a lower percentage of the genes in a predicted target was recorded as being involved in the pathway (3% to 40%, mean 11%), based on albeit incomplete current knowledge of the pathways.

There is one paper [84] which co-cites MED4 and one of its 14 predicted targets, ILF2, where ILF2 is given as one possible candidate for forming the molecular bridge between the Ada-Two-A-containing (ATAC) histone acetyltransferase and Mediator coactivator complexes. For DDX10 there is one paper [85] which co-cites DDX10 and one of its 11 predicted targets, TNFSF14, in a study of changes in hormone receptor target genes and chromatin modifying enzymes after proteasome inhibition in breast cancer cells. There is also one paper [86] which co-cites RIPK2 and one of its 12 predicted targets, EGR1, in a list of genes that are up- or down-regulated in response to the activation of at least one NF-*κ*B family member.

POGZ is cocited with one predicted target SP1 in a paper [87] concerning the proteins that interact with SP1. In a second paper [88] it is cocited with predicted targets CAD, MSH2 and MTA1, all four being identified as SUMO-2 binding proteins. It is cocited with JRK in [89] and [90], and cocited with BRIP1 in a study of gene expression profiling to predict survival in lung squamous cell carcinoma [91].

YWHAZ is cocited with FZD7 in a paper on attention-deficit/hyperactivity disorder [92], with ATXN1 in a paper on the interaction of Akt-Phosphorylated Ataxin-1 with 14-3-3 [93], and with SOS2 in a paper on epidermal growth factor receptor phosphorylation sites [94]. NFATC3 is cocited with IKBKB in a paper on analysis of steady-state nuclear hormone receptor coactivator complexes [95]. E2F5 is cocited with ITGA5 in a paper about miRNA control of tumour cell invasion and metastasis [96].

ADAR has one predicted target, JUNB, and the two are cocited in four papers. In a paper concerning c-Jun Amino-Terminal Kinase-1 mediates glucose-responsive upregulation of ADAR2 in Pancreatic Beta-Cells [97], in a paper on the suppression of the interferon and NF-*κ*B responses by severe fever with thrombocytopenia syndrome virus [98], in a paper on host cell transcription in response to Varicella-Zoster virus infection of human T cells and fibroblasts [99], and in a paper on bacterial pathogens modulating an apoptosis differentiation program in human neutrophils [100].

SMAD5 is cocited with ECT2 in an analysis of novel transcription factor FLJ20420 [101].

## Discussion

In this paper we have investigated the potential for using multiple matched aCGH and expression data sets from cancer samples for inferring gene regulatory relationships. We found genes which show significant aCGH/expression correlation across a large number of the 30 data sets in the study, and found considerable within and between data set consistency for these measurements. Clustering based on between data set consistency appears to reflect the underlying pathologies of the data sets. The study is using cancer data sets as natural knockdown/amplification experiments, rather than investigating cancer genomics per se, but inevitably the analysis is revealing potential driver genes and illustrating both the commonality and the differences in the various pathologies included in the study.

Whilst combining the data sets in a meta-analysis gives a clear and consistent signal of self aCGH/expression correlation for the potential regulators, predicting *trans*-acted targets for these potential regulators is more difficult. Even though the potential regulators investigated show self aCGH/expression correlation in up to 17 of the data sets, the maximum number of data sets which show a significant correlation between a regulator-target aCGH/expression is 6. Part of the problem is experimental noise in the data and possibly also the recognised difficulty of incorrect mappings [102], but the main reason for the problem is likely to be biological. Whilst there is some commonality in regulator-target aCGH/expression, there is also considerable heterogeneity, being specific to tissue type, pathology and experiment. As well as tissue specificity, compensatory pathways and non-linear responses are also likely to be making major contributions to the observed heterogeneity. The outcome of heterogeneity is that the amount of extra information gained from combining data sets is reduced. The type of meta-analysis we have employed is however highly stringent, that is significant relationships are detected only if they are sufficiently significant in enough individual data sets. We adopted this approach in order to investigate the base-line possibilities of the data. Analysis of individual data sets, or a carefully chosen subset of data sets based on pathology, produces far more predictions. For example the meta-analysis does not improve the significance of the experimentally confirmed regulators from our previous study [1] (where the predictions were based on either one experiment or on ten experiments). More detailed analysis of the consistency of regulator-target predictions between the 30 data sets does however suggest that there is more information buried within the data than is apparent from the lists created by taking a threshold of B-H adjusted *p*-values.

Some of the regulator-target predictions are substantiated by published data, although such substantiation is inevitably proscribed by the well-known limitations of current knowledge bases, namely incomplete and inaccurate annotations, low resolution, missing and cell specific information and the dynamic nature of the systems being studied [103].

For computational simplicity we defined potentially *trans*-acting genes as two genes which are located on different chromosomes. Alternatively we could have used the third step of our algorithm, the correlation of a target's aCGH with its regulator's expression to define *trans*-action. High correlation suggests coamplification/codeletion, hence close proximity on the genome. Examining the *p*-values from this step in the algorithm indicates that using this definition would have included on average only an extra 2% of genes in the study.

In general the potential regulators which are known to be transcription factors have more predicted *trans*-acted targets than those potential regulators that are not known to be transcription factors. Some potential regulators that are known to be transcription factors have no predicted *trans*-acted targets, whilst a few have many, POGZ and HSBP1 being the main examples. We observe more targets which have expression negatively correlated with their potential regulator's aCGH, than targets which have expression positively correlated with their potential regulator's aCGH.

Interpretation of the output from matched aCGH/expression studies when these are viewed as large scale gene amplification/deletion experiments is complicated by a number of factors. Some problems are common to conventional knockdown experiments such as cell type variability of a regulatory effect and the occurrence of compensatory regulation. Regulatory effects are tissue specific [28] and specific to a cell's physiological state, with compensatory pathways and potentially a number of regulatory mechanisms affecting expression. Significantly *down* regulated genes can be found in amplified chromosomal regions [18]; one study finding 14% of downregulated genes appearing within regions of DNA gain and 9% of upregulated genes appeared in regions of DNA loss [104]. The main difference between conventional knockdown experiments and inference from matched aCGH/expression studies is that the status of a large number of genes are being changed at the same time. However in our previous study we have shown, through experimental validation, that careful analysis of such data sets can reveal valid gene regulatory relationships [1]. Analysis of matched aCGH/expression data can only reveal a small part of a complex network of gene relationships [18], but we have shown that the predictions from such an analysis can be accurate enough to advise experimental investigation and for incorporating with other data into probabilistic models of gene regulation [1].

The combined data sets are a valuable resource and the regulator-target predictions presented here only include those potential regulators which have significant aCGH/expression correlation in the largest number of data sets. There are many other potential regulators which have significant aCGH/expression correlation in smaller subsets of the data sets, so in future work we plan to provide a simple web application by which researchers can interrogate for themselves the 30 data sets, and subsets of the 30 data sets, for potential regulator and target genes of interest.

## Supporting Information

File S1 **Supporting Information and Figures S1 to S5 and Tables S1 to S2.**
(PDF)

File S2 **Supporting Results, Potential regulators, Sheets S1 and S2.**
(XLS)

File S3 **Supporting Results, Predicted targets, for potential regulators from list of top 30 potential regulators that are not transcription factors, positive regulation.**
(XLS)

File S4 **Supporting Results, Predicted targets, for potential regulators from list of top 30 potential regulators that are not transcription factors, negative regulation.**
(XLS)

File S5 **Supporting Results, Predicted targets, for potential regulators from list of top 72 potential regulators that are transcription factors, positive regulation.**
(XLS)

File S6 **Supporting Results, Predicted targets, for potential regulators from list of top 72 potential regulators that are transcription factors, negative regulation.**
(XLS)

Checklist S1 PRISMA checklist.
(DOC)
