# Supplementary material for: A Meta-Analysis of Multiple Matched Copy Number and Transcriptomics Data Sets for Inferring Gene Regulatory Relationships
Source: PLoS One. 2014 Aug 22;9(8):e105522. doi: 10.1371/journal.pone.0105522 (PMC4141782; doi:10.1371/journal.pone.0105522)
Supplement: File S1 — Supporting Information and Figures S1 to S5 and Tables S1 to S2. (PDF) [file pone.0105522.s001.pdf]

Supplementary Information, Figures and Tables: A meta-analysis of multiple matched copy number and transcriptomics datasets for inferring gene regulatory relationships

Richard Newton*, Lorenz Wernisch, MRC Biostatistics Unit, Cambridge, United Kingdom
∗ e-mail: richard.newton@mrc-bsu.cam.ac.uk

Figure S1: Quantile-Quantile plots for the 30 datasets. Each plot compares the quantiles of the Pearson correlations between a gene' aGCH profile and its expression profile for each gene in the dataset, with the quantiles obtained from two random datasets containing the same number of samples.

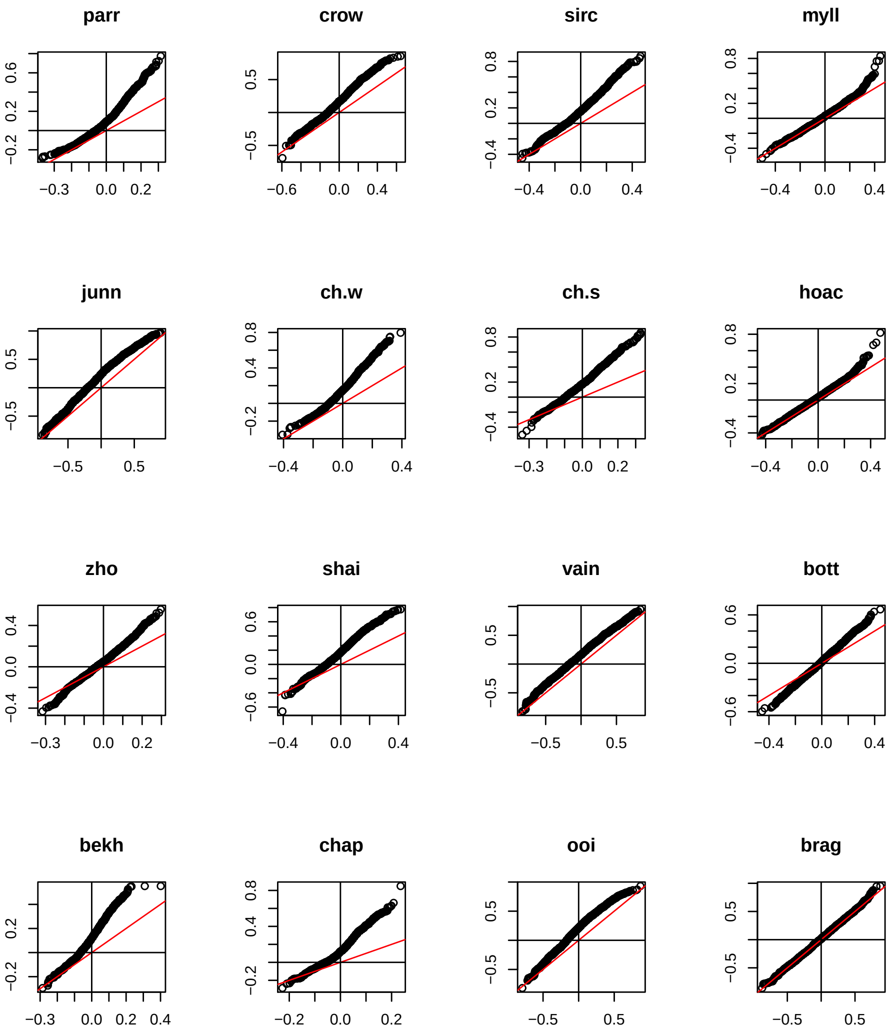

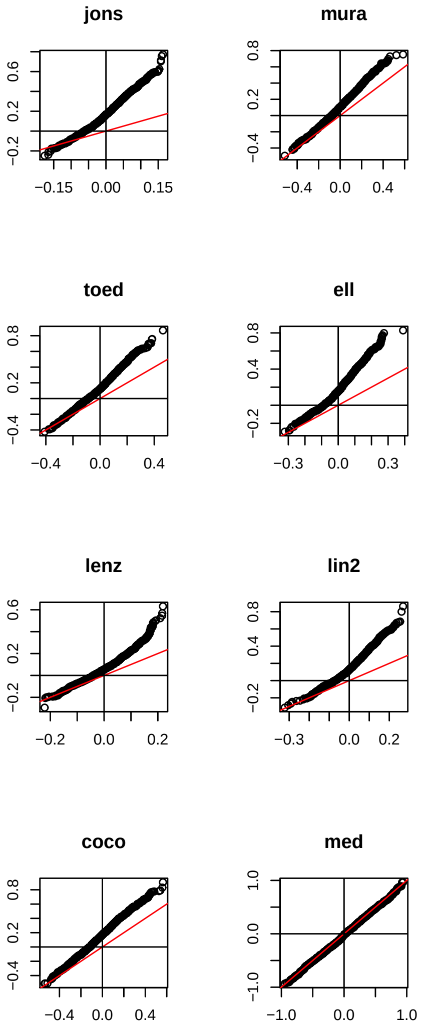
jons
0.6
0.2
−0.2
−0.15
0.00
0.15
mura
0.8
0.2
−0.4
−0.4
0.0
0.4
lin1
0.8
0.2
−0.4
−0.4
0.0
0.4
beck
0.5
−0.5
−0.6
0.0
0.6
toed
0.8
0.2
−0.4
−0.4
0.0
0.4
ell
0.8
0.4
−0.2
−0.3
0.0
0.3
gra.1
0.6
0.0
−0.6
−0.4
0.0
0.4
gra.2
0.5
−0.5
−0.4
0.0
0.4
lenz
0.6
0.2
−0.2
−0.2
0.0
0.2
lin2
0.8
0.4
−0.2
−0.3
0.0
0.2
micc
0.5
−0.5
−0.5
0.5
tayl
0.6
0.2
−0.2
−0.3
0.0
0.2
coco
0.8
0.2
−0.4
−0.4
0.0
0.4
med
1.0
0.0
−1.0
−1.0
0.0
1.0

Assessment of Correlation methods

We investigated the use of four different correlation methods, namely Pearson, Spearman, partial and partial on ranks. Partial correlations were calculated using the R package GeneNet [1,2]. Computational reasons restricted the maximum number of genes for a partial correlation analysis to 15000. So all four methods were applied to a subset of the data restricted to a maximum of 15000 genes. Those data sets with more than this number of genes were reduced in size by taking the 15000 genes with the maximum variance of expression.

The performance of the four correlation methods was assessed and compared using the consistency of predictions from a cross-validation analysis. Each data set was studied independently. A data set was randomly divided into two equal sized cross-validation data sets, and two lists of correlation p-values were calculated from each of these, for all the genes in the data set. The correlation being between a gene's aCGH profile and its expression profile. The genes were ordered by increasing p-value. We then needed to compare the two lists. One approach would be a rank correlation method such as Kendall's τ. The lists we are comparing are however very long (up to 15000 genes) and in practice we are interested in only the top most significant genes, but Kendall's τ places equal weight on the rankings of genes anywhere in the list. We therefore adopted a method which takes the top genes in one list (a gene-set) and looks at their ranks in the second list, and vice-versa. In order to treat all correlation methods and data sets equally in this comparison analysis we took the top genes to be the top s genes in a list in all cases. The size of the gene-set s was arbitrarily chosen to be 10. The Kolmogorov-Smirnov test (R function ks.test) was used to test whether the gene-set derived from the first half of the data set was enriched in the ordered list from the second half of the data set. For each data set this procedure was repeated ten times, that is, on ten random divisions of the data set. And the procedure was carried out for each of the four correlation methods. The result was a mean and range of cross-validation enrichment scores for each data set, for each correlation method. These values were used to compare the consistency of prediction of the different correlation methods. p-values were derived for the enrichment scores by randomising the gene labels of each half data set, after having randomly divided a data set into two equal sized cross-validation data sets, and performing the test as described, and repeating 1000 times in order to generate a null distribution of enrichment scores.

Figure S2 plots the average enrichment score for each data set using Pearson correlation against the average obtained using Spearman correlation. The plot indicates Pearson and Spearman correlation give similar results, but Spearman gives slightly greater consistency for a majority of the data sets. This would be expected since Spearman, being a ranked based correlation is less sensitive to outliers in the data. Figure S3 plots the average enrichment scores obtained using partial correlation against the averages obtained using Spearman correlation. The plot indicates Spearman correlation gives greater consistency than partial correlation. The analysis was repeated using partial correlation on ranks which gave similar results to the partial correlation results.

References

1. Schäfer J, Strimmer K (2005) A shrinkage approach to large-scale covariance matrix estimation and implications for functional genomics. Stat Appl Genet Mol Biol 4: 32.

2. Schäfer J, Opgen-Rhein R, Strimmer K. Genenet: Modelling and inferring gene networks. URL `http://cran.r-project.org/web/packages/GeneNet`.

Figure S2: Plot of the average cross-validation enrichment score for each dataset using Pearson correlation against the same using Spearman correlation. Filled circles denote datasets with more than 30 samples.

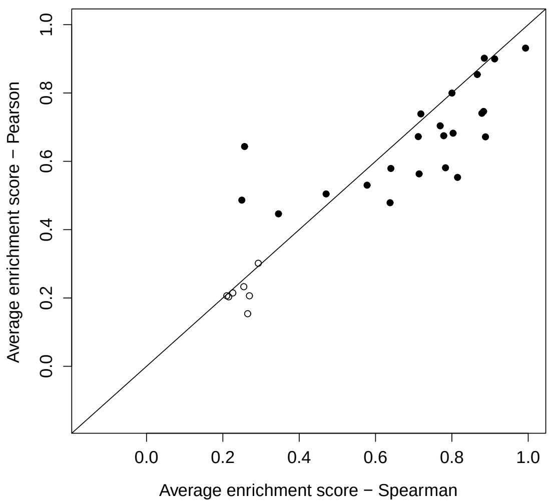

Figure S3: Plots of the average cross-validation enrichment scores for each dataset using partial correlation against the same using Spearman correlation. Filled circles denote datasets with more than 30 samples.

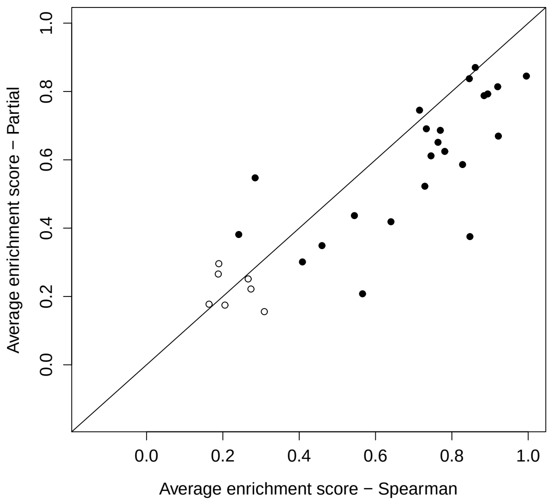

Figure S4: Clustering datasets according to aCGH variance of genes. The top 10 genes from one dataset were used as a gene-set and scored for enrichment in the second half, and vice-versa. The two enrichment scores were averaged and this value minus one used as a distance measure for clustering, using Ward's method. The nine datasets with low within dataset consistency (see Figure 1 main text) were excluded from the clustering (pr=prostate, lg=lung, oa=oesophageal, ly=lymphoma, bl=bladder, br=breast, ne=neuroblastoma, pl=pleural, ps=myeloma, pn=pancreas, ga=gastric, bn=glioma).

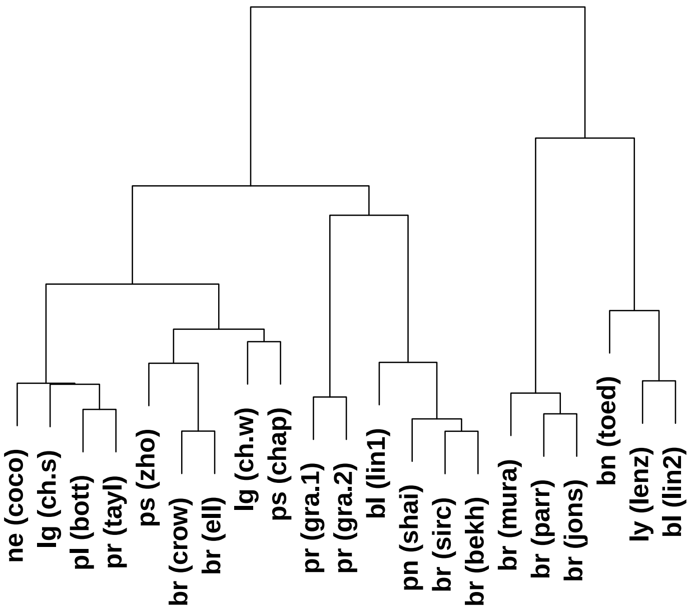

Figure S5: Continuation of Figure 5c & 5d, for each potential regulator which are transcription factors (numbers 31 to 72), bar charts showing the number of predicted targets at a significance level of 0.05 (red) and 0.1 (blue) a. positive regulation b. negative regulation.

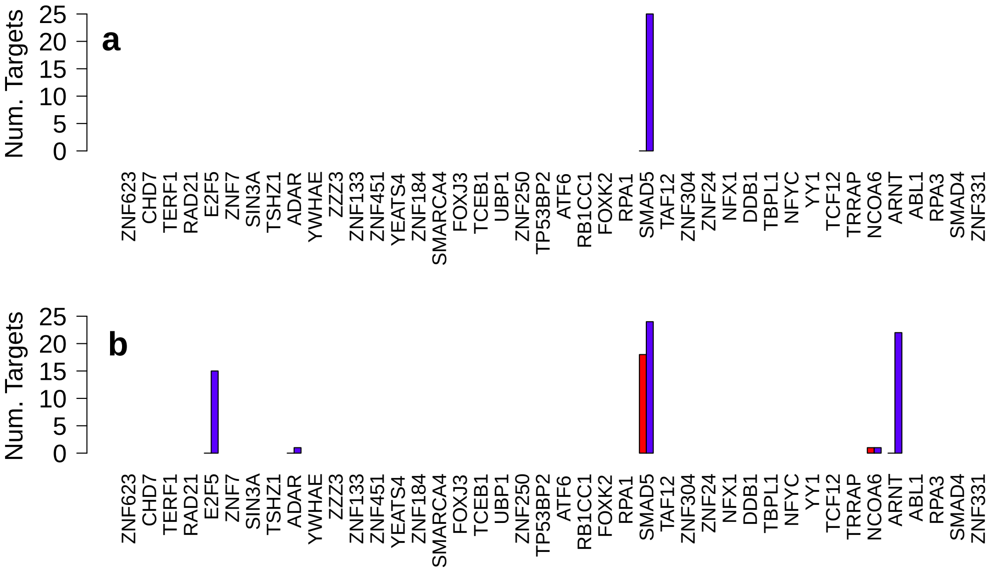

Table S1. Top 30 potential regulators - not transcription factors, based on the Spearman correlation of a gene's aCGH with its expression, summarising the pathologies in which a gene shows significant correlation

Gene	Chr	Locus	N	breast	prostate	lung	urothelial	gastric	lymphoma	neuroblastoma	myeloma	glioma	leiomyosarcoma	oesophageal	mesothelioma	pancreas	vulva
				7	4	3	2	2	2	2	2	1	1	1	1	1	1
				7	3	2	2	1	1	1	2	1	0	1	1	1	0
PCM1	8	22-p	17	6	2	2	2	0	1	1	2	0	0	0	0	1	0
ELP3	8	21.1p	17	5	3	2	2	0	1	1	2	0	0	0	0	1	0
MED4	13	14.12q	17	6	3	1	2	0	1	0	2	1	0	0	0	1	0
MCPH1	8	23.1p	16	6	2	2	2	0	1	0	2	0	0	0	0	1	0
COPS3	17	11.2p	16	6	1	2	2	0	1	1	2	0	0	0	0	1	0
PREP	6	22q	15	6	2	2	1	0	1	0	2	0	0	0	0	1	0
DDX10	11	22-q	15	6	1	2	1	0	1	0	2	1	0	0	0	1	0
BCL9	1	21q	15	7	1	2	2	0	0	0	2	1	0	0	0	0	0
CDC16	13	34q	15	5	2	2	2	0	0	0	2	1	0	0	0	1	0
HDAC2	6	21q	15	6	1	2	2	0	0	1	2	0	0	0	0	1	0
AZIN1	8	21.3q	15	7	1	2	2	0	1	1	1	0	0	0	0	0	0
SS18L1	20	13.3q	14	6	1	2	2	0	0	0	2	0	0	0	0	1	0
TGDS	13	32.1q	14	5	1	2	2	1	0	0	2	0	0	0	0	1	0
YTHDF1	20	13.33q	14	7	1	2	2	0	0	0	1	0	0	0	0	1	0
COG2	1	42.2q	14	6	0	2	2	0	1	0	2	0	0	0	0	1	0
PPP2R2A	8	21.2p	14	5	1	2	2	0	1	0	2	0	0	0	0	1	0
PTDSS1	8	22q	14	6	2	2	2	0	0	0	1	0	0	0	0	1	0
AKAP11	13	14.11q	14	5	2	2	2	0	0	0	2	0	0	0	0	1	0
IKBKB	8	11.2p	14	6	1	2	2	0	0	0	2	0	0	0	0	1	0
MBTPS1	16	24q	14	4	3	2	2	0	0	0	2	0	0	0	0	1	0
UCHL3	13	21.33q	14	5	2	1	2	0	1	0	2	0	0	0	0	1	0
AARS	16	22q	14	5	2	2	2	0	0	0	2	0	0	0	0	1	0
ATXN10	22	13q	14	6	0	2	2	0	0	0	2	1	0	0	0	1	0
RAF1	3	25p	14	6	0	2	2	0	1	1	1	0	0	0	0	1	0
PPP3CC	8	21.3p	14	5	2	2	1	0	0	0	2	1	0	0	0	1	0
TBCE	1	42.3q	14	6	0	2	1	0	1	1	2	0	0	0	0	1	0
RIPK2	8	21q	14	5	2	2	1	0	1	0	2	0	0	0	0	1	0
INTS6	13	14.3q	14	6	1	2	2	0	1	0	2	0	0	0	0	0	0
UBAP2	9	11.2p	14	5	1	2	2	0	1	0	2	0	0	0	0	1	0
GNA12	7	22.3p	14	5	1	2	2	0	1	0	1	1	0	0	0	1	0

Chr = Chromosome, Locus = Gene locus, N = number of datasets with significant correlation (B-H adjusted p-value < 0.05), the first row immediately below pathologies gives total number of datasets with that pathology, the following row gives the total number of datasets with more than 30 samples with that pathology.

Table S2. Top 30 potential regulators - transcription factors, based on the Spearman correlation of a gene's aCGH with its expression, summarising the pathologies in which a gene shows significant correlation

Gene	Chr	Locus	N	breast	prostate	lung	urothelial	gastric	lymphoma	neuroblastoma	myeloma	glioma	leiomyosarcoma	oesophageal	mesothelioma	pancreas	vulva
				7	4	3	2	2	2	2	2	1	1	1	1	1	1
				7	3	2	2	1	1	1	2	1	0	1	1	1	0
GTF2F2	13	14q	16	6	3	1	2	0	0	0	2	1	0	0	0	1	0
TAF2	8	24q	14	7	1	2	1	0	1	0	1	0	0	0	0	1	0
SETDB1	1	21q	14	6	1	2	2	0	0	0	2	0	0	0	0	1	0
ELF1	13	13q	14	4	2	2	2	0	1	0	2	0	0	0	0	1	0
YWHAZ	8	22.3q	13	4	2	2	2	0	1	0	1	0	0	0	0	1	0
PARP1	1	41-q	13	5	1	2	1	0	1	0	2	1	0	0	0	0	0
ACTL6A	3	26.33q	13	5	0	2	2	0	1	0	2	0	0	0	0	1	0
PSMB1	6	27q	13	5	0	2	2	0	1	0	1	1	0	0	0	1	0
SMARCA2	9	24.3p	13	4	0	2	2	0	1	0	2	1	0	0	0	1	0
NCOR1	17	11.2p	13	5	0	2	2	0	1	0	2	0	0	0	0	1	0
MAP3K7	6	15q	13	5	2	1	2	0	1	0	1	0	0	0	0	1	0
HSBP1	16	23.3q	12	4	3	2	0	0	0	0	2	0	0	0	0	1	0
SMARCE1	17	21.2q	12	6	0	2	1	0	0	0	1	1	0	0	0	1	0
POGZ	1	21.1q	12	5	1	1	2	0	1	0	1	0	0	0	0	1	0
RCOR3	1	32.3q	12	5	0	2	2	0	0	0	2	0	0	0	0	1	0
TRIM33	1	13.1p	12	6	0	1	1	0	1	0	2	0	0	0	0	1	0
ARID4B	1	42.1-q	12	6	0	2	2	0	0	0	1	0	0	0	0	1	0
MNAT1	14	23q	12	4	0	2	2	0	1	0	2	0	0	0	0	1	0
NFATC3	16	22q	12	5	1	2	1	0	0	0	2	0	0	0	0	1	0
TBP	6	27q	12	6	1	1	1	0	1	0	1	0	0	0	0	1	0
AATF	17	12q	12	6	1	2	1	0	0	0	1	0	0	0	0	1	0
SMAD2	18	21q	12	3	1	2	1	0	1	0	2	1	0	0	0	1	0
AP2B1	17	11.2-q	12	5	0	2	1	0	1	0	2	0	0	0	0	1	0
SNAPC3	9	22.3p	12	4	1	2	1	0	1	0	2	1	0	0	0	0	0
SNW1	14	22.1-q	12	3	1	2	2	0	0	1	2	0	0	0	0	1	0
SMARCC1	3	21.31p	12	5	1	1	1	0	1	1	1	0	0	0	0	1	0
HSF2	6	22q	12	5	1	1	1	0	1	0	2	0	0	0	0	1	0
PSIP1	9	22.2p	12	4	0	2	2	0	1	0	2	0	0	0	0	1	0
RB1	13	14.2q	12	5	1	1	2	0	0	0	2	1	0	0	0	0	0
CREBBP	16	13.3p	12	5	1	2	1	0	0	1	1	0	0	0	0	1	0

Chr = Chromosome, Locus = Gene locus, N = number of datasets with significant correlation (B-H adjusted p-value < 0.05), the first row immediately below pathologies gives total number of datasets with that pathology, the following row gives the total number of datasets with more than 30 samples with that pathology.
